# Cardiovascular and metabolic morbidity in women with previous gestational diabetes mellitus: a nationwide register-based cohort study

**DOI:** 10.1186/s12933-022-01609-2

**Published:** 2022-09-09

**Authors:** Maria Hornstrup Christensen, Katrine Hass Rubin, Tanja Gram Petersen, Ellen Aagaard Nohr, Christina Anne Vinter, Marianne Skovsager Andersen, Dorte Moeller Jensen

**Affiliations:** 1 Steno Diabetes Center Odense, Odense University Hospital, Kloevervaenget 10, 5th floor, 5000 Odense, Denmark; 2 Department of Gynecology and Obstetrics, Odense University Hospital, Kloevervaenget 23, 2nd Floor, 5000 Odense, Denmark; 3 Department of Clinical Research, University of Southern Denmark, J.B. Winsloewsvej 19, 3rd Floor, Odense, Denmark; 4 Research Unit OPEN, Department of Clinical Research, University of Southern Denmark, J.B. Winsloewsvej 9A, 3rd Floor, Odense, Denmark; 5 OPEN-Open Patient Data Explorative Network, Odense University Hospital, Heden 16, Ground Floor, 5000 Odense, Denmark; 6 Department of Endocrinology, Odense University Hospital, J.B. Winsloewsvej 4, 5000 Odense, Denmark

**Keywords:** Cardiovascular and metabolic risk, Cohort study, Epidemiology, Gestational diabetes mellitus, Pregnancy, Pregnancy complications, Register-based study

## Abstract

**Background:**

Gestational diabetes mellitus (GDM) is associated with adverse pregnancy outcomes and has maternal health implications reaching beyond the perinatal period. We aimed to investigate the incidence and severity of cardiovascular and metabolic morbidity in women with previous GDM in a Danish population and to study whether proxies of impaired beta cell function—insulin treatment during GDM pregnancy and development of subsequent manifest diabetes mellitus—influence incident risk of cardiovascular and metabolic morbidity.

**Methods:**

A nationwide register-based cohort study was conducted on the complete cohort of 700,648 women delivering in Denmark during 1997–2018. The exposure variable was GDM and primary outcome was overall cardiovascular and metabolic morbidity. Secondary outcomes were major cardiovascular disease (ischemic heart disease, heart failure, and/or stroke/transient cerebral ischemia), hypertension, dyslipidemia, and venous thrombosis. Severity of morbidity was assessed using number of hospital contacts with diagnosis codes related to cardiovascular and metabolic morbidity and number of redemptions of prescribed medication related to cardiovascular and metabolic morbidity in women who developed cardiovascular and metabolic morbidity after pregnancy.

**Results:**

The median follow-up period was 10.2–11.9 years with a total range of 0–21.9 years. GDM was associated with increased risk of any cardiovascular and metabolic morbidity (adjusted HR 2.13 [95% CI 2.07–2.20]), major cardiovascular disease (adjusted HR 1.69 [95% CI 1.55–1.84]), hypertension (adjusted HR 1.89 [95% CI 1.82–1.96], dyslipidemia (adjusted HR 4.48 [95% CI 4.28–4.69]), and venous thrombosis (adjusted HR 1.32 [95% CI 1.16–1.50]). Insulin treatment during pregnancy and subsequent development of manifest diabetes exacerbated the risk estimates. Previous GDM was associated with more hospital contacts and more redeemed prescriptions in women developing cardiovascular and metabolic morbidity (p < 0.001).

**Conclusions:**

Previous GDM was associated with significantly higher risk of cardiovascular and metabolic morbidity, especially incident dyslipidemia. Risks were exacerbated by proxies of beta cell impairment. Severity of morbidity was significantly worse if GDM preceded cardiovascular and metabolic morbidity.

## Background

Gestational diabetes mellitus (GDM) develops when the maternal insulin secretion is insufficient to meet increased insulin demand during pregnancy. Worldwide GDM prevalence ranges from 1% to ≥ 30%, depending on screening procedures, diagnostic criteria and population characteristics [1]. In Denmark, the prevalence is 3% [2]. GDM is associated with increased risk of obstetric complications, including hypertensive disorders, cesarean section, and preterm delivery [1]. GDM treatment comprises diet and exercise counselling and pharmacological treatments, including insulin treatment, if blood glucose targets are not met [3].

Women with GDM have a tenfold increased risk of later development of manifest diabetes mellitus, mainly type 2 diabetes [4]. Diabetes and insulin resistance are associated with increased risk of cardiovascular disease (CVD), which is a main contributor to increased morbidity and leading cause of mortality [5–7]. Recently, a systematic review and meta-analysis reported a twofold risk of incident CVD in women with previous GDM, with the association persisting irrespective of subsequent type 2 diabetes [8]. Therefore, strong evidence of an association between GDM per se and CVD is mounting. It is clinically relevant to elucidate whether the severity of morbidity in women who eventually develop CVD is affected by previous GDM. To our knowledge, no study has addressed this subject. Cardiovascular and metabolic morbidity (CVMM) are intertwined [9]. Albeit, to our knowledge, no previous studies have investigated dyslipidemia as a metabolic component of the cardiovascular morbidity spectrum after GDM. Additionally, whether insulin treatment during GDM pregnancy is associated with CVMM risk remains unclear. Insulin treatment in pregnancy may indicate a more profound insulin resistance and/or impaired beta cell function, and thus may potentially be associated with higher risk.

This study sought to investigate long-term CVMM in Danish women following a GDM diagnosis, based on data from national registries on the total population of women delivering in Denmark during 1997–2018. The study investigated CVMM incidence and severity in the Danish population of women with previous GDM. Furthermore, the study investigated whether proxies of impaired beta cell function—insulin treatment during GDM pregnancy and development of manifest diabetes mellitus—influence incident CVMM risk.

## Methods

### Study design and data sources

This study was a nationwide register-based cohort study. In Denmark, register data on all individuals are collected prospectively and are stored in administrative registries. All permanent residents of Denmark have unique identification numbers; thus individual-leveled linkage of data across registries is possible [10–12].

We obtained historical data from several registries provided by Statistics Denmark and The Danish Health Data Authority. From the Danish Medical Birth Register, which contains data on pregnancies and live- and stillbirths in Denmark, the population of delivering women was identified, and pregnancy and delivery data were provided [13]. The Danish National Patient Registry provided data on ICD-10 diagnosis codes (primary and secondary) of hospital contacts [14], whereas The Danish National Prescription Register provided data on redemptions of prescribed medication [15]. Additionally, demographic and socioeconomic data were collected from relevant registers [16–18].

### Study population

All women giving birth in Denmark from January 1, 1997 to December 31, 2018 were identified. The study unit was women and each woman could contribute with ≥ 1 delivery during the study period. Index date was the date of conception in index pregnancy (first pregnancy in the study period). We excluded women with preexisting diabetes and/or preexisting CVD at or up to 2 years before the index date based on selected diagnosis codes and/or medication (Table 1). The time span of 2 years was necessitated to secure identical exclusion criteria for all included women regardless of time of study entry as we had data from 1995 onwards and as our study period commenced in 1997. Additionally, we excluded women with missing data on selected sociodemograhic covariates identified a priori (age, parity, marital status, ethnicity, income, occupation, education).

**Table 1:** Definitions of variables according to ICD-10 codes and anatomical therapeutic chemical groups

	ICD-10 codes or ATC groups
*Exclusion criteria*	
Preexisting diabetes	ICD-10: E10-E14, O240-O243, O245, O249 and/or ATC: A10 (except A10BA02) (≥ 2 redemptions)
Preexisting cardiovascular disease (CVD)	ICD-10: I00-I09, I20-I99, G45-G46 and/or ATC: C01, B01 (≥ 2 redemptions)
*Exposure*	
Gestational diabetes mellitus (GDM)	ICD-10: O244
*Outcomes*	
Any cardiovascular and metabolic morbidity defined as any of:	
Dyslipidemia	ICD-10: E78 (and no preexisting dyslipidemia (see below))
Hypertension	ICD-10: I10-I15 (and no preexisting hypertension (see below))
Ischemic heart disease	ICD-10: I20-I25
Pulmonary embolism	ICD-10: I26
Cardiac arrhythmia	ICD-10: I48, I490
Heart failure	ICD-10: I50
Stroke/transient cerebral ischemia (TCI)	ICD-10: I61, I63-I66, G45-G46
Other venous thrombosis	ICD-10: I80-I82
Antithrombotic agents	ATC: B01 (≥ 2 redemptions)
Lipid modifying agents	ATC: C10 (≥ 2 redemptions)
Antihypertensive agents	ATC: C02-C03, C07-C09 (≥ 2 redemptions)
Major CVD defined as any of:	
Ischemic heart disease	ICD-10: I20-I25
Heart failure	ICD-10: I50
Stroke/TCI	ICD-10: I61, I63-I66, G45-G46
Hypertension	ICD-10: I10- I15 *and/or* ATC: C02-C03, C07-C09 (≥ 2 redemptions) *and* no preexisting hypertension (see below)
Dyslipidemia	ICD-10: E78 and/or ATC: C10 (≥ 2 redemptions) and no preexisting dyslipidemia (see below)
Venous thrombosis	ICD-10: I26, I80-I82
*Potential confounders*	At or 2 years before index date
Preexisting dyslipidemia	ICD-10: E78 and/or ATC: C10 (≥ 2 redemptions)
Preexisting hypertension	ICD-10: I10-I15 (from 2 years before index date until gestational week 20) *and/or* ATC: C02-C03, C07-C09 (≥ 2 redemptions from 2 years before index date until gestational week 20)
Preexisting polycystic ovary syndrome	ICD-10: E282
Preexisting hirsutism	ICD-10: L680
Preexisting metformin	ATC: A10BA02 (≥ 2 redemptions)
*Risk factors for outcome*	In index pregnancy unless stated otherwise
Preeclampsia	ICD-10: O14 (after gestational week 20)
Gestational hypertension	ICD-10: O13, O16 (after gestational week 20)
*Potential intermediate covariates*	
Insulin treatment (in any GDM pregnancy)	ICD-10: O244E and/or ATC: A10A (≥ 1 redemption in ≥ 1 GDM pregnancy)
Incident subsequent diabetes prior to outcome	ICD-10: E10-E14, O240-O243, O245, O249 and/or ATC: A10 (≥ 2 redemptions) and no incident outcome prior to incident diabetes

### Exposure

Exposure was GDM defined as GDM diagnosis code (Table 1) in ≥ 1 pregnancy during the study period; unexposed women had GDM in zero pregnancies. As each woman may have both GDM and non-GDM pregnancies during the study period, GDM was treated as a time-varying exposure, thereby facilitating correct exposure status of each pregnancy.

In Denmark, selective GDM screening is performed based on individual risk factor assessment. GDM is diagnosed if the 2-h venous plasma glucose value at a 75 g oral glucose tolerance test is ≥ 9 mmol/L [19].

### Outcomes

Outcomes were incident CVMM categories based on selected diagnosis codes and/or medical treatment (Table 1). To qualify medication as indicative of morbidity, ≥ 2 redemptions of prescribed medication were required.

The primary outcome was incidence of any CVMM, defined as diagnosis codes of ischemic heart disease, heart failure, stroke/TCI, hypertension, venous thrombosis, dyslipidemia, cardiac arrhythmia, and/or medication (antihypertensive, antithrombotic, and/or lipid modifying agents). Secondary outcomes were incident major CVD (diagnosis codes of ischemic heart disease, heart failure, and/or stroke/transient cerebral ischemia (TCI)), hypertension (diagnosis code and/or medication), venous thrombosis (diagnosis code), and dyslipidemia (diagnosis code and/or medication). Each woman could experience ≥ 1 outcome event, thus may be represented in ≥ 1 outcome category.

Morbidity severity was investigated in women with incidence of any CVMM. As proxy for severity, we investigated number of additional hospital contacts with diagnosis codes and number of additional redemptions of prescribed medication contained within the any CVMM definition. Numbers were examined within 3 months, 1 year, and 3 years after date of first hospital contact or redemption. Furthermore, we generated a mortality variable (death of any cause after date of first CVMM).

### Follow-up and risk time

Follow-up commenced 6 weeks after delivery date in index pregnancy. The scope of this study was incidence of long-term and not pregnancy-related outcomes, and thus follow-up was initiated at the end of the postpartum period.

Risk time commenced at start of follow-up and continued until first occurrence of outcome, death, emigration, or end of study. However, in case of multiple pregnancies during the study period, risk time during any subsequent pregnancy from the date of conception to 6 weeks postpartum was excluded from the total risk time in order to limit the risk time to the non-pregnant state. Risk time was categorized as exposed or unexposed depending on presence or absence of GDM, respectively. However, due to the nature of GDM and the CVMM outcomes, it was decided, that it was only possible to contribute with exposed risk time after GDM exposure, regardless of potential, subsequent non-GDM pregnancies: thus once exposed, always exposed regarding categorization of risk time.

### Covariates

Based on existing literature and a directed acyclic graph, potential confounders were identified a priori. Data on these covariates were obtained from the index pregnancy as baseline data (Tables 1 and 2).

Potential confounders were maternal age, parity (as a time-varying confounder), pregestational BMI, smoking in pregnancy, ethnicity, marital status, income in the calendar year prior to delivery, highest level of completed education, occupation, calendar year of delivery, preexisting hypertension, dyslipidemia, polycystic ovary syndrome (PCOS)/hirsutism, metformin treatment, and finally comorbidity as expressed by the Charlson Comorbidity Index (CCI) [20].

Proxies of beta cell impairment were investigated for their potential influence on CVMM risk. These proxies were insulin treatment during GDM and subsequent development of manifest diabetes after pregnancy but prior to outcome (Table 1).

### Statistical analyses

For descriptive statistics, Wilcoxon rank sum and chi-squared tests were used for non-normally distributed continuous and categorical variables, respectively. Associations between GDM and outcomes were analyzed using Cox regression models. In considering non-proportional hazards, we included interaction terms between study period and age, parity, and index year. Morbidity severity was investigated using Wilcoxon rank sum test and logistic regression.

The influence of proxies of beta cell impairment on CVMM risk was investigated by including ‘insulin treatment during GDM pregnancy’ and ‘subsequent diabetes prior to outcome’ as time-varying covariates in interaction terms with GDM in the adjusted Cox regression models. Additionally, in a separate analysis, only ‘subsequent diabetes’ was included as an interaction term for the outcomes any CVMM and major CVD, thereby generating risk estimates disregarding treatment modality of GDM and solely examining the influence of subsequent diabetes on the risk. We used likelihood ratio test to compare models with and without interaction terms.

Missing data on gestational age at delivery (< 2%) was handled by imputing the mean gestational age. Extensive missing data on pregestational BMI was expected as registration in the Danish Medical Birth Register was not initiated until late 2003; likewise regarding registration of smoking during pregnancy which commenced in late 1997 [13]. Therefore, these covariates were not included in the main adjusted analysis but were handled in sensitivity analyses.

All statistical analyses were performed using STATA 16 software (StataCorp, College Station, TX). P-values < 0.05 were considered significant.

### Sensitivity analyses

Several sensitivity analyses were performed. To minimize risk of GDM misclassification, we restricted the study population to (1) women without previous deliveries prior to the study period, and (2) women with GDM diagnosis code registered after gestational week 20 and absence of diagnosis code for pregestational diabetes after the index date during the same pregnancy. To address potential bias due to exclusion of women with missing data on the selected demographic and socioeconomic data and due to imputation regarding missing data on gestational age at delivery, we generated missing categories for each confounder instead of excluding the women, and we excluded women with missing data on gestational age at delivery instead of imputation, respectively. Thereafter, we included BMI and smoking in the adjusted analyses and performed additional sensitivity analyses excluding women with potential risk factors for CVMM present at or during the index pregnancy (preeclampsia, gestational hypertension, preterm delivery, stillbirth, cesarean section, preexisting hypertension, dyslipidemia, PCOS/hirsutism, and metformin treatment). Finally, an analysis was performed in which we commenced follow-up at index date and refrained from excluding risk time contributed from index/conception date to 6 weeks postpartum in subsequent pregnancies, thus facilitating insight into incidence of both pregnancy-related and long-term outcomes.

## Results

### Study population

**Fig. 1:**
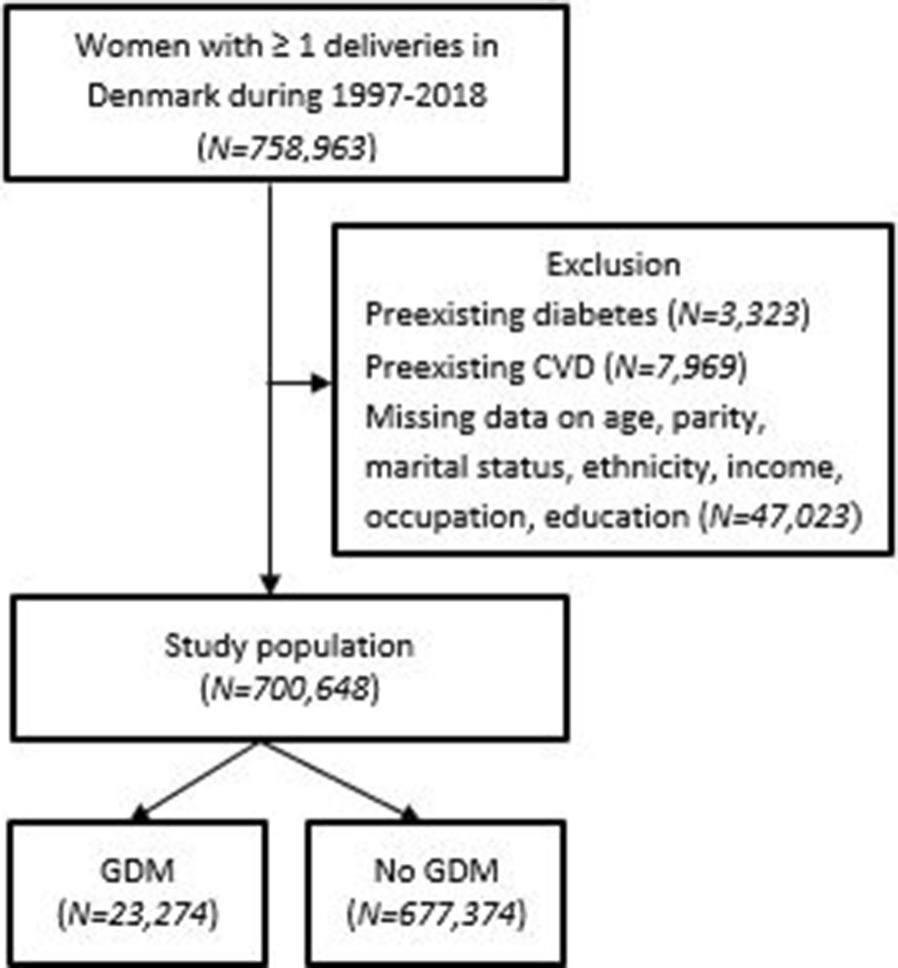
Flowchart of study population. *CVD* cardiovascular disease, *GDM* gestational diabetes mellitus

During 1997–2018, 758,963 women had ≥ 1 delivery. We excluded women with preexisting diabetes (*n* = 3323), preexisting CVD (*n* = 7969), and missing data on the a priori chosen confounders (*n* = 47,023). The final study population comprised 700,648 women; 23,274 (3.3%) women with and 677,374 (96.7%) women without GDM (Fig. 1). In women with GDM, 12.9% were treated with insulin.

A total of 697,173 women entered the survival analyses, as women experiencing outcomes from the index date to 6 weeks postpartum were censored prior study entry, as they were not considered as being at risk. Follow-up time differed among outcome categories, with medians ranging from 10.2 (IQR 4.6–16.3) years to 11.9 (IQR 5.4–11.9) years; total range was 0–21.9 years. Cumulative incidence of diabetes from follow-up initiation to first occurrence of outcome, death, emigration, or end of study in women with and without previous GDM was 16.1% and 1.4%, respectively.

### Baseline data

Baseline data from the index pregnancy are shown in Table 2. Women with GDM had higher BMI and were more likely to have preexisting comorbidities; to be of non-Western origin or descendant; to be in the lowest or highest income groups; to have a lower educational level; and have lower employment rate than women without GDM. Obstetric complications were more prevalent in women with GDM.

**Table 2:** Baseline characteristics from index pregnancy according to gestational diabetes mellitus (GDM)

	Total (*N* = 700,648)	GDM (*N* = 23,274)	No GDM (*N* = 677,374)	p-values^*^
*Clinical baseline characteristics*				
Age, years	28 (25–31)	28 (25–32)	28 (25–31)	< 0.001
Primiparity	546,257 (78.0)	19,607 (84.2)	526,650 (77.8)	< 0.001
Pregestational BMI, kg/m^2 a^	23 (21–26)	27 (23–32)	23 (21–26)	< 0.001
Underweight (< 18.5)	16,691 (4.5)	309 (2.0)	16,382 (4.6)	< 0.001
Normalweight (18.5–24.9)	240,285 (64.1)	5443 (34.5)	234,842 (65.4)	< 0.001
Overweight (25–29.9)	75,599 (20.2)	4750 (30.2)	70,849 (19.7)	< 0.001
Obesity (≥ 30)	42,042 (11.2)	5255 (33.4)	36,787 (10.3)	< 0.001
Smoking during pregnancy^b^	105,493 (17.4)	3666 (17.3)	101,827 (17.4)	0.719
Preexisting hypertension	8672 (1.2)	552 (2.4)	8120 (1.2)	< 0.001
Preexisting dyslipidemia	959 (0.1)	78 (0.3)	881 (0.1)	< 0.001
Preexisting PCOS/hirsutism	4605 (0.7)	417 (1.8)	4188 (0.6)	< 0.001
Preexisting use of metformin	5608 (0.8)	647 (2.8)	4961 (0.7)	< 0.001
Charlson comorbidity score of 0	693,815 (99.0)	22,949 (98.6)	670,866 (99.0)	< 0.001
*Demographic characteristics*				
Ethnicity				
Danish	615,422 (87.8)	18,483 (79.4)	596,939 (88.1)	< 0.001
Immigrant, Western	21,923 (3.1)	617 (2.7)	21,306 (3.2)	< 0.001
Immigrant, Non-Western	52,346 (7.5)	3585 (15.4)	48,761 (7.2)	< 0.001
Descendant	10,957 (1.6)	589 (2.5)	10,368 (1.5)	< 0.001
Single/not living with a partner	86,098 (12.3)	2808 (12.1)	83,290 (12.3)	0.291
Income				
Low tertile	220,188 (31.4)	7606 (32.7)	212,582 (31.4)	< 0.001
Middle tertile	239,690 (34.2)	7398 (31.8)	232,292 (34.3)	< 0.001
High tertile	240,770 (34.4)	8270 (35.5)	232,500 (34.3)	< 0.001
Highest completed education				
Lower secondary	136,628 (19.5)	5565 (23.9)	131,063 (19.4)	< 0.001
Upper secondary	294,433 (42.0)	9697 (41.7)	284,736 (42.0)	0.260
Post secondary	269,587 (38.5)	8012 (34.4)	261,575 (38.6)	< 0.001
Occupation				
Employed	503,167 (71.8)	16,016 (68.8)	487,151 (71.9)	< 0.001
Unemployed/welfare payment	91,627 (13.1)	2,645 (11.4)	88,982 (13.1)	< 0.001
Under education	70,836 (10.1)	2,977 (12.8)	67,859 (10.0)	< 0.001
Early retirement	3342 (0.5)	255 (1.1)	3087 (0.5)	< 0.001
*Obstetric characteristics*				
Preeclampsia	25,129 (3.6)	1612 (6.9)	23,517 (3.5)	< 0.001
Gestational hypertension	11,820 (1.7)	924 (4.0)	10,896 (1.6)	< 0.001
Gestational age at delivery, days	280 (273–287)	277 (268–283)	280 (273–287)	< 0.001
Preterm delivery^c^	47,915 (6.8)	2378 (10.2)	45,537 (6.7)	< 0.001
Stillbirth	2813 (0.4)	200 (0.9)	2613 (0.4)	< 0.001
Cesarean section	136,854 (19.5)	6601 (28.4)	130,253 (19.2)	< 0.001

Data presented as median (IQR) or n (%)

*BMI* body mass index, *PCOS* polycystic ovary syndrome

^*^GDM vs. no GDM

^a^N = 374,617

^b^N = 606,281

^c^Delivery before gestational week 37

### Previous GDM and incident cardiovascular and metabolic morbidity

Table 3 shows incidence rates and crude and adjusted hazard ratios (HRs) of outcomes, according to previous GDM. All risk estimates were significantly higher in women with previous GDM. For any CVMM, a 2.1-fold adjusted risk was found, whereas the adjusted risk for major CVD was 1.7-fold. The highest aHR was found for dyslipidemia (4.48 [95% CI 4.28–4.69]) and the lowest for venous thrombosis (1.32 [95% CI 1.16–1.50]). For the subcategory outcome cardiac arrhythmia, significance was lost in the adjusted analysis (data not shown).

**Table 3:** Risk of cardiovascular and metabolic morbidity according to preceding gestational diabetes mellitus (GDM)

	GDM			No GDM			HR (95% CI)^a^	
	N events	Risk time^b^	IR (95% CI)	N events	Risk time^b^	IR (95% CI)	Crude	Adjusted^c^
Any CVMM	4202	152,321	27.59 (26.76–28.43)	92,318	7,170,185	12.88 (12.79–12.96)	2.17 (2.10–2.23)	2.13 (2.07–2.20)
Major CVD	562	183,312	3.07 (2.82–3.33)	12,208	7,839,093	1.56 (1.53–1.59)	1.97 (1.81–2.14)	1.69 (1.55–1.84)
Ischemic heart disease	319	184,764	1.73 (1.55–1.93)	6174	7,879,454	0.78 (0.76–0.80)	2.20 (1.96–2.46)	1.79 (1.60–2.01)
Heart failure	59	186,765	0.32 (0.24–0.41)	918	7,914,654	0.12 (0.11–0.12)	2.77 (2.13–3.60)	2.26 (1.73–2.95)
Stroke/TCI	231	185,690	1.24 (1.09–1.42)	5777	7,881,218	0.73 (0.71–0.75)	1.70 (1.49–1.93)	1.53 (1.34–1.74)
Hypertension	2866	167,369	17.12 (16.51–17.76)	70,030	7,530,085	9.30 (9.23–9.37)	1.84 (1.77–1.91)	1.89 (1.82–1.96)
Venous thrombosis	235	185,138	1.27 (1.12–1.44)	7077	7,866,033	0.90 (0.88–0.92)	1.40 (1.23–1.60)	1.32 (1.16–1.50)
Dyslipidemia	2185	172,507	12.67 (12.15–13.21)	19,930	7,802,375	2.55 (2.52–2.59)	5.20 (4.97–5.43)	4.48 (4.28–4.69)

*CVMM* cardiovascular and metabolic morbidity, *CVD* cardiovascular disease, *TCI* transient cerebral ischemia

^a^No GDM as reference

^b^Risk time presented in person years and incidence rates (IR) presented as number of events per 1000 person years

^c^Adjusted for age, parity, preexisting PCOS/hirsutism/hypertension/dyslipidemia, Charlson Comorbidity Index, ethnicity, marital status, income, education, occupation, and calendar year of delivery

All but one sensitivity analysis resulted in similar risk estimates as the main analyses; inclusion of BMI and smoking in the adjusted analysis resulted in minor attenuation of the estimates and loss of significance for venous thrombosis (aHR 0.85 [95% CI 0.67–1.07]).

### Insulin treatment in GDM pregnancy, subsequent diabetes, and incident cardiovascular and metabolic morbidity

**Fig. 2:**
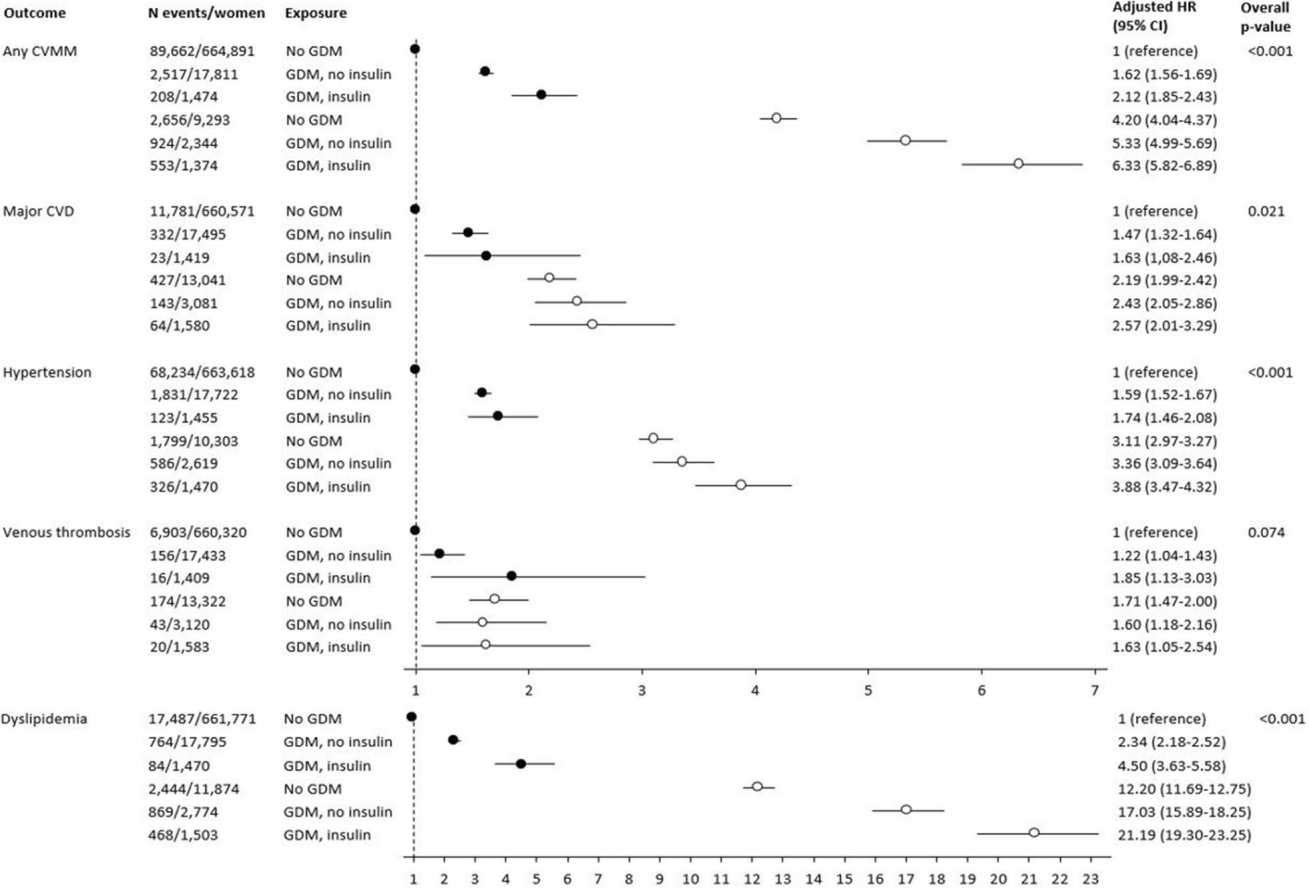
Cardiovascular and metabolic morbidity according to preceding GDM, insulin-treated GDM, and subsequent diabetes. Forest plot of hazard ratios for cardiovascular and metabolic morbidity adjusted for age, parity, preexisting PCOS/hirsutism/hypertension/dyslipidemia, Charlson comorbidity score, ethnicity, marital status, income, education, occupation, and calendar year of delivery. Reference group was women with no GDM and without subsequent diabetes. Black circles = women without subsequent diabetes; white circles = women with subsequent diabetes. *GDM* gestational diabetes mellitus, *CVMM* cardiovascular and metabolic morbidity, *CVD* cardiovascular disease

Figure 2 shows the adjusted HRs for CVMM outcomes when the study population was categorized into six mutually exclusive exposure groups according to GDM, insulin treatment in GDM pregnancy, and development of subsequent diabetes. The reference group was women without GDM and without subsequent diabetes. The figure shows a pattern of increasing HRs for outcomes when preceded by insulin-treated GDM, compared to non-insulin-treated GDM and non-GDM pregnancy. This pattern was observed in women with and without subsequent diabetes. The patterns were similar, and the overall p-values were significant for all outcome categories, except for venous thrombosis.

**Fig. 3:**
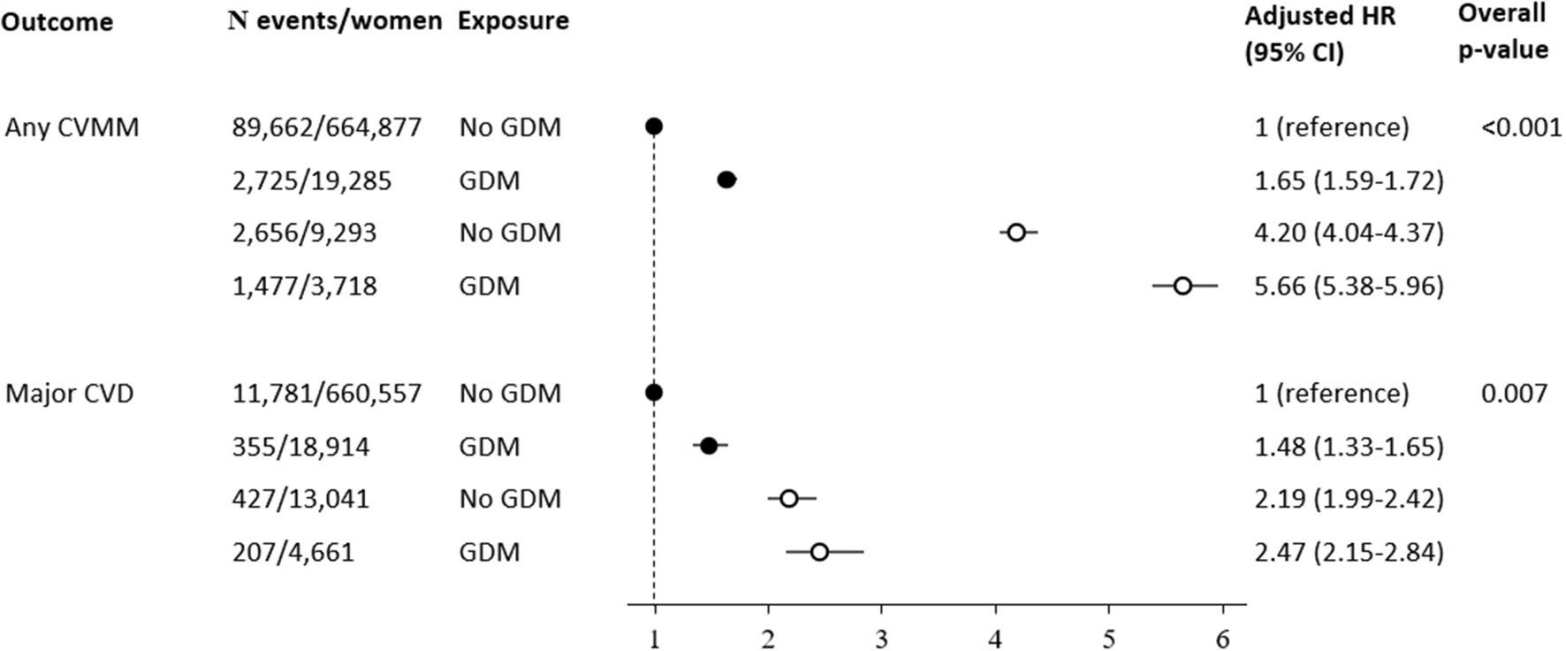
Cardiovascular and metabolic morbidity according to preceding GDM and subsequent diabetes. Forest plot of hazard ratios for cardiovascular and metabolic morbidity adjusted for age, parity, preexisting PCOS/hirsutism/hypertension/dyslipidemia, Charlson comorbidity score, ethnicity, marital status, income, education, occupation, and calendar year of delivery. Reference group was women with no GDM and without subsequent diabetes. Black circles = women without subsequent diabetes; white circles = women with subsequent diabetes. *GDM* gestational diabetes mellitus, *CVMM* cardiovascular and metabolic morbidity, *CVD* cardiovascular disease

Results on overall effect of GDM (thus disregarding treatment modality) on risk of any CVMM and major CVD in women with and without subsequent diabetes are shown in Fig. 3. The reference group was women without GDM and without subsequent diabetes. Adjusted HR for any CVMM was 1.65 [95% CI 1.59–1.72] in women with GDM and no subsequent diabetes; 4.20 [95% CI 4.04–4.37] in women without GDM and with subsequent diabetes, whereas it was 5.66 [95% CI 5.38–5.96] in women with GDM and with subsequent diabetes. For major CVD, the corresponding risk estimates were 1.48 [95% CI 1.33–1.65], 2.19 [95% CI 1.99–2.42], and 2.47 [95% CI 2.15–2.84].

### Severity of cardiovascular and metabolic morbidity

In women with incidence of any CVMM, previous GDM was associated with older age at index pregnancy (30.3 vs. 29.4 years, p = 0.002) and at first outcome event (39.6 vs. 39.4 years, p < 0.001). Previous GDM was not associated with mortality (adjusted odds ratio 0.9 [95% CI 0.7–1.1]).

Table 4 shows that compared to women without GDM, the number of hospital contacts with CVMM diagnosis codes was higher in women with previous GDM within 3 years after initial hospital contact (p < 0.001), but not within 1 year (p = 0.196). Number of CVMM redemptions was higher in women with previous GDM within 1 year, as well as within 3 years, after initial redemption (p < 0.001). No differences were observed within 3 months (data not shown).

**Table 4:** Severity of morbidity according to preceding gestational diabetes mellitus (GDM)

	GDM (*N* = 4,232)	No GDM (*N* = 92,604)	p-value	OR (95% CI)^a^	
				Crude	Adjusted^b^
*Hospital contacts*^c^	*N* = 1742	*N* = 34,733			
Within 1 year	0 (0–1)	0 (0–1)	0.196		
0	994 (57.1)	20,589 (59.3)		1 (reference)	1 (reference)
1–2	574 (33.0)	10,775 (31.0)		1.10 (0.99–1.23)	1.13 (1.02–1.26)
≥ 3	174 (10.0)	3369 (9.7)		1.07 (0.91–1.26)	1.11 (0.94–1.31)
Within 3 years	1 (0–2)	0 (0–1)	< 0.001		
0	854 (49.0)	18,890 (54.4)		1 (reference)	1 (reference)
1–2	609 (35.0)	11,139 (32.1)		1.21 (1.09–1.35)	1.24 (1.11–1.38)
3–4	180 (10.3)	3040 (8.8)		1.31 (1.11–1.55)	1.34 (1.14–1.59)
≥ 5	99 (5.7)	1664 (4.8)		1.32 (1.06–1.63)	1.37 (1.10–1.70)
*Redemptions*^d^	*N* = 3951	*N* = 83,087			
Within 1 year	3 (0–5)	3 (0–5)	< 0.001		
0–1	1173 (29.7)	30,815 (37.1)		1 (reference)	1 (reference)
2–3	1224 (31.0)	23,268 (28.0)		1.38 (1.27–1.50)	1.42 (1.31–1.55)
4–6	818 (20.7)	16,292 (19.6)		1.32 (1.20–1.45)	1.42 (1.29–1.56)
≥ 7	736 (18.6)	12,712 (15.3)		1.52 (1.38–1.67)	1.61 (1.46–1.78)
Within 3 years	7 (2–12)	5 (0–11)	< 0.001		
0–1	716 (18.1)	22,294 (26.8)		1 (reference)	1 (reference)
2–5	957 (24.2)	21,266 (25.6)		1.40 (1.27–1.55)	1.37 (1.24–1.51)
6–9	741 (18.8)	12,849 (15.5)		1.80 (1.62–1.99)	1.87 (1.68–2.08)
10–13	627 (15.9)	11,234 (13.5)		1.74 (1.56–1.94)	1.92 (1.71–2.15)
≥ 14	910 (23.0)	15,444 (18.6)		1.83 (1.66–2.03)	1.97 (1.77–2.20)

Morbidity severity in women with incident cardiovascular and metabolic morbidity

Data presented as median (IQR) or n (%)

^a^Odds ratio, reference group: No GDM and 0 additional hospital contacts or 0–1 additional redemption, respectively

^b^Adjusted for age, parity, preexisting PCOS/hirsutism/hypertension/dyslipidemia, Charlson Comorbidity Index, ethnicity, marital status, income, education, occupation, and calendar year of delivery

^c^Number of additional hospital contacts with diagnosis codes within ischemic heart disease, heart failure, stroke/TCI, hypertension, dyslipidemia, venous thrombosis, and/or cardiac arrhythmia

^d^Number of additional redemptions within antithrombotic, lipid modifying and/or antihypertensive agents

## Discussion

This study showed that previous GDM was associated with significantly higher risk of all cardiovascular and metabolic outcomes and that the association was exacerbated by insulin treatment in GDM pregnancy in women with and without subsequent diabetes. Moreover, we found that previous GDM was associated with more severe morbidity in women with incident CVMM. The GDM prevalence of 3.3% was expected considering the study period, the study population, and the definition of study unit (women rather than pregnancies).

### Previous GDM and incident cardiovascular and metabolic morbidity

Consistent with previous findings, our findings indicated that our overall any CVMM-composite was significantly higher in women with previous GDM than in women without GDM. We found a more than twofold increased risk for any CVMM after GDM. The systematic review and meta-analysis by Kramer et al. showed that the crude, relative risk of CVD was nearly twofold in women with previous GDM and almost 1.6-fold after adjustment for various confounders [8]. The studies included in the meta-analysis had diverse outcome definitions, mostly comprising composites. The risk estimates were significantly heterogeneous but the combined crude risk estimate approximated that of our any CVMM-composite. A recent Danish register study found a 1.4-fold increased adjusted risk for overall CVD in women with previous GDM [21]. This estimate was less than the 2.1-fold increased adjusted risk for any CVMM found in our study. This discrepancy might be explained by the inclusion of medical treatment to identify CVMM and of the metabolic component of dyslipidemia in our overall outcome.

The inclusion of dyslipidemia added important new knowledge regarding a clinically relevant metabolic component of future cardiovascular-related morbidity after GDM. To our knowledge, dyslipidemia as a separate outcome in association with previous GDM had not been explored previously. We found that the adjusted risk for incident dyslipidemia was 4.5-fold in women with previous GDM. For hypertension, the adjusted risk was almost twofold. Other studies have also investigated hypertension as a separate outcome and found a significant association [21–23]. We defined hypertension and dyslipidemia as outcomes per se, albeit they represent independent risk factors for other CVMMs [9]. Thus, our finding of the remarkably increased risk of incident dyslipidemia after GDM is particularly of clinical importance.

Our results on major CVD were consistent with results from other research teams internationally. Major CVD constituted grave cardiovascular conditions (ischemic heart disease, heart failure, and stroke/TCI), and albeit being of low incidence in absolute numbers, it is remarkable that in our study, previous GDM was associated with a nearly 70% increased risk for major CVD. For heart failure specifically, the risk was more than doubled after adjustment for confounders. The latter result corresponded with findings from the Danish study [21] and two Canadian studies [24, 25] but contrasted with findings from a US study [26]. Regarding an association between GDM and stroke, the evidence is insufficient according to a scoping review from 2014 [27]. Three studies have recently contributed with results indicating no association [22, 23, 26]. Two of these studies were large cohort studies like ours and had study populations of approximately 1.5 million and 850,000 women [23, 26]. Contrarily, we found a 53% significantly increased adjusted risk of stroke/TCI in women with previous GDM. The discrepancy between our results and their results is potentially explained by the differences in follow-up periods after delivery; they had shorter total follow-up periods (upper range 7 and 1 year) than our study (upper range 21.9 years). Our data supported findings from the other Danish register study [21].

As stated previously, GDM screening in Denmark is selective and based on presence of risk factors. These risk factors may contribute to the poorer cardiovascular and metabolic outcome after pregnancy in women with previous GDM and potentially our results could be impacted by the screening strategy. Yet, in general our findings correspond to those reported in studies from countries with universal GDM screening, e.g. the US [26] and Canada [24, 28, 29].

We conducted several sensitivity analyses, thereby addressing known CVMM risk factors during index pregnancies. All sensitivity analyses resulted in similar, hence significant, risk estimates as the main analysis, except for venous thrombosis, as described previously. The association between previous GDM and incident cardiovascular and metabolic outcomes identified in our study was plausibly robust and not explained by other known risk factors. Additionally, our study revealed that the increased CVMM risk was evident during pregnancy and the immediate postpartum period, and thus not restricted to long-term.

### Insulin treatment in GDM pregnancy, subsequent diabetes, and incident cardiovascular and metabolic morbidity

To our knowledge, the association between insulin treatment during previous GDM pregnancy and incident CVMM risk had not been investigated previously. We found that insulin treatment in GDM pregnancy was associated with a higher risk of CVMM outcomes than no insulin treatment. For example, in women without subsequent diabetes prior to outcome, a 30% increase in incident any CVMM risk was observed among women with previous insulin-treated GDM, compared to women with non-insulin-treated GDM. For the outcome dyslipidemia, the corresponding risk was almost doubled. Additionally, the pattern of increased risk in association with insulin treatment was observed in women with subsequent diabetes. Our study suggests that beta cell impairment represented by insulin treatment in GDM pregnancy seemed to increase the CVMM risk, irrespective of subsequent diabetes prior to CVMM. However, the relative influence of insulin treatment seemed to be of smaller impact in women with subsequent diabetes. The proportion of women with insulin treatment was lower than anticipated based on experience from clinical practice. Most likely the number was underestimated; however, the expected consequence of this would be more conservative risk estimates and thus the overall conclusions regarding the impact of insulin treatment during GDM pregnancy were deemed valid and robust.

The associations between GDM and future diabetes [4, 30] and between diabetes and future CVD [5, 6] are well described. According to the systematic review and meta-analysis by Kramer et al., some studies have found conflicting results as to whether the observed association between GDM and CVD was due to subsequent diabetes [8]. Similar to other studies [21, 23, 24, 26, 29], our study found a significant association, regardless of subsequent diabetes. When restricting to women without subsequent diabetes prior to CVMM, previous GDM was associated with nearly 1.7-fold increased risk of any CVMM and an almost 1.5-fold increased risk of major CVD.

Thus, our study showed that the two proxies of beta cell impairment influenced the association between GDM and CVMM by exacerbating risk. Moreover, we found that in women with incident diabetes, previous GDM seemed to be an additional risk factor per se for development of CVMM.

### Severity of cardiovascular and metabolic morbidity

To our knowledge, the severity of morbidity in women with incident CVMM according to previous GDM had not been investigated previously. Women with preceding GDM exhibited significantly worse cardiovascular and metabolic health within 3 years after initial hospital contact/redemption. Within 1 year, this was only evident concerning medication and within 3 months, no differences were found. This finding indicated that the worse risk profile was not of an immediate matter upon recognition of CVMM but rather had a trajectory that was characterized by a slower, yet still significant progression.

In Denmark, women with GDM are recommended a follow-up on glycemic status every 1–3 years [19]. This potential surveillance bias may lead to a potential overestimation of risk in women with previous GDM, as compared to risk in women without GDM. However, as adherence to the recommendation is poor [31], the effect of this bias may be limited. Women with previous GDM were, on the other hand, possibly more likely to be diagnosed at an earlier point in their disease trajectory due to the recommended follow-ups, leading to timely initiation of management and treatment. This could theoretically explain that despite worse health, no difference in all-cause mortality was found between women with and without previous GDM. In women with incident CVMM, we found statistically significant differences in age at index pregnancy and at first CVMM according to history of GDM; however in clinical practice these differences seem negligible.

### Strengths and limitations

Our study had several strengths. This very large study was based on the complete cohort of delivering women in Denmark and had a follow-up period of up to 21.9 years. Data were derived from registers containing information on all residents in Denmark. Thus, due to the nature of the study, selection and information bias were minimized. Additionally, the registers are considered valid and of high quality for research, hence providing opportunity for reliable results [11, 14, 15]. Likewise, the validity of the GDM diagnosis code in the Danish registers is reassuringly high [32]. We could account for each woman’s complete history on GDM during the study period, thus reducing exposure misclassification risk. Regarding outcomes, the validity of CVMM diagnosis code is generally high [33]. Furthermore, the combined use of diagnosis codes and redemptions of prescriptions as outcomes improved the study’s clinical relevance. For insulin treatment during GDM pregnancy, data on diagnosis code for insulin-treated GDM and redemption of prescribed insulin during GDM pregnancy were combined to increase depiction of this variable. We addressed important potential confounders and mediators and had an extensive analytical strategy with several sensitivity analyses; thus, our results seem valid and robust.

The study had some limitations. In general, register-based studies hold the limitation that the original data were neither collected for research purposes nor collected by the researcher. Regarding exclusion criteria, it would have been optimal to exclude women with any history of preexisting diabetes and/or CVD and not only within the past 2 years prior to index date. Albeit, in general the Danish population of fertile women is relatively young and healthy so only very limited potential impact of this is expected. Regarding outcomes, several other cardiovascular and metabolic disorders could have been relevant to investigate, however the message of an increased cardiovascular and metabolic risk after GDM is supported well by the selected outcomes. The long study period implies potential changes in screening procedures, diagnostic criteria, and management. The Danish GDM guidelines were revised in 2003 based on a Danish study [34, 35]. However, the diagnostic criteria only changed marginally and the variations may not affect our conclusions significantly. GDM misclassification risk was another limitation. Due to selective GDM screening in Denmark, GDM exposure was likely underestimated. Additionally, some women may be misclassified as non-GDM but potentially develop GDM in future pregnancies. However, these misclassifications were only expected to produce more conservative results. The number of women treated with insulin during GDM was likely underestimated. This was partly due to the diagnosis code registration practice but also that some women requiring insulin might be supplied with the first insulin pens without prescription from the hospital, and thus would not be identified by redemptions. This underestimation may potentially attenuate CVMM risk related to insulin treatment, and thus underestimate the true risk difference associated with insulin treatment of GDM as it is expected to give rise to more conservative results. Despite sound confounder control, we expect some residual confounding. Additionally, some eligible confounders, including smoking, were self-reported, and thus potentially underreported. Another limitation was due to our use of data on confounders and intermediate covariates from the index pregnancy. Whereas few covariates, including ethnicity, were static, most of these, including smoking, BMI and occupation, were dynamic, and may not be representative later in life. Furthermore, subsequent diabetes was not differentiated into subtypes, and finally the pragmatic choice of number of hospital contacts and redemptions of prescribed medication as proxy for morbidity severity might not capture severity sufficiently.

## Conclusion

In conclusion, significant associations existed between GDM and incidence of cardiovascular and metabolic outcomes. The associations were exacerbated by insulin treatment during GDM pregnancy in women with and without subsequent diabetes mellitus. Additionally, previous GDM was associated with worse cardiovascular and metabolic health in women with incident CVMM. Thus, our study strengthens the perception of GDM per se as risk factor for future cardiovascular and metabolic health. Of particular clinical importance is our novel finding of a remarkably increased risk for incident dyslipidemia after GDM, which contains additional perspectives for future management, preventive strategies, and actions after pregnancy.

## Data Availability

According to Danish legislation concerning personalized data, the dataset used for this study is not publicly available. Authorization of access to the data has been granted restrictively to the authors by the Danish National Health Data Authority upon specific application, however other researchers are equally eligible to apply for access to data.

## Abbreviations

ATC: Anatomical therapeutic chemical classification system
BMI: Body mass index
CCI: Charlson Comorbidity Index
CVD: Cardiovascular disease
CVMM: Cardiovascular and metabolic morbidity
GDM: Gestational diabetes mellitus
ICD-10: International Classification of Diseases (10th revision)
PCOS: Polycystic ovary syndrome
TCI: Transient cerebral ischemia

## References

[1] McIntyre HD, Catalano P, Zhang C, Desoye G, Mathiesen ER, Damm P (2019). Gestational diabetes mellitus. Nat Rev Dis Primers.

[2] Jeppesen C, Maindal HT, Kristensen JK, Ovesen PG, Witte DR (2017). National study of the prevalence of gestational diabetes mellitus among Danish women from 2004 to 2012. Scand J Public Health.

[3] Egan AM, Dunne FP (2019). Optimal management of gestational diabetes. Br Med Bull.

[4] Vounzoulaki E, Khunti K, Abner SC, Tan BK, Davies MJ, Gillies CL (2020). Progression to type 2 diabetes in women with a known history of gestational diabetes: systematic review and meta-analysis. BMJ.

[5] Glovaci D, Fan W, Wong ND (2019). Epidemiology of diabetes mellitus and cardiovascular disease. Curr Cardiol Rep.

[6] Nelson AJ, Peterson ED, Pagidipati NJ (2019). Atherosclerotic cardiovascular disease and heart failure: determinants of risk and outcomes in patients with diabetes. Prog Cardiovasc Dis.

[7] Global Disease Burden 2013 Mortality and Causes of Death Collaborators. Global, regional, and national age-sex specific all-cause and cause-specific mortality for 240 causes of death, 1990–2013: a systematic analysis for the Global Burden of Disease Study 2013. Lancet. 2015;385(9963):117–71. 10.1016/S0140-6736(14)61682-2 PMC4340604 25530442

[8] Kramer CK, Campbell S, Retnakaran R (2019). Gestational diabetes and the risk of cardiovascular disease in women: a systematic review and meta-analysis. Diabetologia.

[9] World Health Organization. Cardiovascular diseases (CVDs). 2021.

[10] Thygesen LC, Daasnes C, Thaulow I, Bronnum-Hansen H (2011). Introduction to Danish (nationwide) registers on health and social issues: structure, access, legislation, and archiving. Scand J Public Health.

[11] Thygesen LC, Ersboll AK (2014). When the entire population is the sample: strengths and limitations in register-based epidemiology. Eur J Epidemiol.

[12] Schmidt M, Schmidt SAJ, Adelborg K, Sundboll J, Laugesen K, Ehrenstein V (2019). The Danish health care system and epidemiological research: from health care contacts to database records. Clin Epidemiol.

[13] Bliddal M, Broe A, Pottegard A, Olsen J, Langhoff-Roos J (2018). The Danish Medical Birth Register. Eur J Epidemiol.

[14] Schmidt M, Schmidt SA, Sandegaard JL, Ehrenstein V, Pedersen L, Sorensen HT (2015). The Danish National Patient Registry: a review of content, data quality, and research potential. Clin Epidemiol.

[15] Pottegard A, Schmidt SAJ, Wallach-Kildemoes H, Sorensen HT, Hallas J, Schmidt M (2017). Data resource profile: The Danish National Prescription Registry. Int J Epidemiol.

[16] Schmidt M, Pedersen L, Sorensen HT (2014). The Danish Civil Registration System as a tool in epidemiology. Eur J Epidemiol.

[17] Jensen VM, Rasmussen AW (2011). Danish education registers. Scand J Public Health.

[18] Baadsgaard M, Quitzau J (2011). Danish registers on personal income and transfer payments. Scand J Public Health.

[19] Dansk Selskab for Obstetrik og Gynækologi (The Danish Society of Obstetrics and Gynaecology). Gestationel diabetes mellitus (GDM). Screening og diagnose 2014. http://gynobsguideline.dk/wp/wp-content/uploads/2013/02/GDM-Sandbjerg-2014-godkendt-2014.pdf.

[20] Quan H, Sundararajan V, Halfon P, Fong A, Burnand B, Luthi JC (2005). Coding algorithms for defining comorbidities in ICD-9-CM and ICD-10 administrative data. Med Care.

[21] Yu Y, Soohoo M, Sørensen HT, Li J, Arah OA (2022). Gestational diabetes mellitus and the risks of overall and type-specific cardiovascular diseases: a population- and sibling-matched cohort study. Diabetes Care.

[22] Daly B, Toulis KA, Thomas N, Gokhale K, Martin J, Webber J (2018). Increased risk of ischemic heart disease, hypertension, and type 2 diabetes in women with previous gestational diabetes mellitus, a target group in general practice for preventive interventions: a population-based cohort study. PLoS Med.

[23] Goueslard K, Cottenet J, Mariet AS, Giroud M, Cottin Y, Petit JM (2016). Early cardiovascular events in women with a history of gestational diabetes mellitus. Cardiovasc Diabetol.

[24] McKenzie-Sampson S, Paradis G, Healy-Profitos J, St-Pierre F, Auger N (2018). Gestational diabetes and risk of cardiovascular disease up to 25 years after pregnancy: a retrospective cohort study. Acta Diabetol.

[25] Echouffo-Tcheugui JB, Guan J, Retnakaran R, Shah BR. Gestational diabetes and incident heart failure: a cohort study. Diabetes Care. 2021. 10.2337/dc21-0552 PMC8929190 34385145

[26] Savitz DA, Danilack VA, Elston B, Lipkind HS (2014). Pregnancy-induced hypertension and diabetes and the risk of cardiovascular disease, stroke, and diabetes hospitalization in the year following delivery. Am J Epidemiol.

[27] Archambault C, Arel R, Filion KB (2014). Gestational diabetes and risk of cardiovascular disease: a scoping review. Open Med.

[28] Kaul P, Savu A, Nerenberg KA, Donovan LE, Chik CL, Ryan EA (2015). Impact of gestational diabetes mellitus and high maternal weight on the development of diabetes, hypertension and cardiovascular disease: a population-level analysis. Diabet Med.

[29] Retnakaran R, Shah BR (2017). Role of type 2 diabetes in determining retinal, renal, and cardiovascular outcomes in women with previous gestational diabetes mellitus. Diabetes Care.

[30] You H, Hu J, Liu Y, Luo B, Lei A (2021). Risk of type 2 diabetes mellitus after gestational diabetes mellitus: a systematic review and meta-analysis. Indian J Med Res.

[31] Nielsen KK, Kapur A, Damm P, de Courten M, Bygbjerg IC (2014). From screening to postpartum follow-up—the determinants and barriers for gestational diabetes mellitus (GDM) services, a systematic review. BMC Pregnancy Childbirth.

[32] Olsen SF, Houshmand-Oeregaard A, Granstrom C, Langhoff-Roos J, Damm P, Bech BH (2017). Diagnosing gestational diabetes mellitus in the Danish National Birth Cohort. Acta Obstet Gynecol Scand.

[33] Sundboll J, Adelborg K, Munch T, Froslev T, Sorensen HT, Botker HE (2016). Positive predictive value of cardiovascular diagnoses in the Danish National Patient Registry: a validation study. BMJ Open.

[34] Jensen DM, Mølsted-Pedersen L, Beck-Nielsen H, Westergaard JG, Ovesen P, Damm P (2003). Screening for gestational diabetes mellitus by a model based on risk indicators: a prospective study. Am J Obstet Gynecol.

[35] Jensen DM, Damm P, Sørensen B, Mølsted-Pedersen L, Westergaard JG, Korsholm L (2003). Proposed diagnostic thresholds for gestational diabetes mellitus according to a 75-g oral glucose tolerance test. Maternal and perinatal outcomes in 3260 Danish women. Diabet Med..

